# Effects of Atypical Antipsychotics, Clozapine, Quetiapine and Brexpiprazole on Astroglial Transmission Associated with Connexin43

**DOI:** 10.3390/ijms22115623

**Published:** 2021-05-25

**Authors:** Kouji Fukuyama, Motohiro Okada

**Affiliations:** Department of Neuropsychiatry, Division of Neuroscience, Graduate School of Medicine, Mie University, Tsu 514-8507, Japan; k-fukuyama@clin.medic.mie-u.ac.jp

**Keywords:** clozapine, quetiapine, brexpiprazole, astrocyte, hemichannel, connexin43

## Abstract

Recently, accumulating preclinical findings suggest the possibility that functional abnormalities of tripartite synaptic transmission play important roles in the pathophysiology of schizophrenia and affective disorder. Therefore, to explore the novel mechanisms of mood-stabilizing effects associated with tripartite synaptic transmission, the present study determined the effects of mood-stabilizing antipsychotics, clozapine (CLZ), quetiapine (QTP) and brexpiprazole (BPZ), on the astroglial l-glutamate release and expression of connexin43 (Cx43) in the astroglial plasma membrane using cortical primary cultured astrocytes. Neither acute (for 120 min) nor subchronic (for 7 days) administrations of CLZ, QTP and BPZ affected basal astroglial l-glutamate release, whereas both acute and subchronic administration of CLZ, QTP and BPZ concentration-dependently enhanced astroglial l-glutamate release through activated hemichannels. Subchronic administration of therapeutic-relevant concentration of valproate (VPA), a histone deacetylase inhibiting mood-stabilizing antiepileptic drug, enhanced the stimulatory effects of therapeutic-relevant concentration of CLZ, QTP and BPZ on astroglial l-glutamate release through activated hemichannel. Subchronic administration of therapeutic-relevant concentration of CLZ, QTP and BPZ did not affect Cx43 protein expression in the plasma membrane during resting stage. After subchronic administration of VPA, acute and subchronic administration of therapeutic-relevant concentrations of CLZ increased Cx43 protein expression in the plasma membrane. Both acute administrations of therapeutic-relevant concentrations of QTP and BPZ did not affect, but subchronic administrations enhanced Cx43 protein expression in the astroglial plasma membrane. Furthermore, protein kinase B (Akt) inhibitor suppressed the stimulatory effects of CLZ and QTP, but did not affect Cx43 protein expression in the astroglial plasma membrane. These results suggest that three mood-stabilizing atypical antipsychotics, CLZ, QTP and BPZ enhance tripartite synaptic glutamatergic transmission due to enhancement of astroglial Cx43 containing hemichannel activities; however, the Cx43 activating mechanisms of these three mood-stabilizing antipsychotics were not identical. The enhanced astroglial glutamatergic transmission induced by CLZ, QTP and BPZ is, at least partially, involved in the actions of these three mood-stabilizing antipsychotics.

## 1. Introduction

Modulations of various monoamine receptors have provided the development of a number of atypical antipsychotics for the treatment of schizophrenia and affective disorders [1,2,3], whereas approximately two-thirds of patients with schizophrenia and affective disorders lack to achieve an adequate response to first-choice pharmacotherapy using conventional atypical antipsychotics, and ultimately as many as one-third of patients remain unwell even after several adequate trials of antipsychotics [1,2,3,4,5]. Therefore, a number of psychiatrists and pharmacologists have been exploring the novel therapeutic strategies associated without monoaminergic hypothesis for the treatment of patients with schizophrenia and affective disorders [1,2,3,6]. Recent neuropharmacological studies suggest that functional abnormalities of tripartite synaptic transmission possibly contribute to pathophysiology of schizophrenia, affective disorder, epilepsy and their associated cognitive impairments [7,8,9,10,11,12,13,14,15,16,17,18,19,20,21,22]. Tripartite synaptic transmission is consisted with exocytotic and non-exocytotic gliotransmitter releases through vesicle, hemichannel and reversely transporter of astrocytes [2,6,8,9,11,12,13,14,15,16,17,18,20]. Connexon is formed by six connexins unit [2,3], and two connexons in two neighboring cells, neurons, astrocytes, oligodendrocytes and microglia form gap-junction with aqueous pore and charged surface walls, whereas single connexon contributes to chemical connection between intra- and extracellular spaces as a hemichannel [2,3,22]. During the resting stage, astroglial gap-junctions are permeable intracellular molecules via functionally opening probability, whereas astroglial hemichannel exhibits low opening probability [2,3,8,22,23,24]. Therefore, it has been considered that astroglial hemichannels are not functional at the resting stages due to their low opening probabilities [23,24]. Contrary to resting stage, under the several pathological conditions, such as depolarization, ischemia, specific cation mobilization and phosphorylation, generate persistent hemichannel opening, resulting in the sustained astroglial non-exocytotic release of excitatory l-glutamate, d-serine, adenosine triphosphate, kynurenine metabolites and eicosanoids, which toxically affect homeostasis systems [2,3,8,9,10,11,12,17,19,20,21,25].

Contrary to toxic function of astroglial hemichannel, recent several studies indicated the possibilities that hemichannel activity are probably involved in the physiological function regarding the regulation of cognition/perception and mood/emotion [2,3,26,27,28,29]. Activated astroglial hemichannels can continue to be persisted for over several hours [8,10,22]. Therefore, even if recovers to neuronal resting stage after hyperexcitability, astrocytes can remain to release non-exocytotic gliotransmitter through activated astroglial hemichannels [2,3,10,11,12,13]. Connexin43 (Cx43) is one of the most widely and predominant expressed isoform in the astrocyte, myocardia and pulmonary cells [2,3]. Postmortem studies revealed downregulation of Cx43 expression in the locus coeruleus, frontal cortex, mediodorsal thalamic nucleus and caudate nucleus of patients with mood disorders compared to healthy individuals [2,30,31,32,33]. Interestingly, these Cx43 downregulation regions coincide with regulation regions of cognition/perception and emotion/mood [3,6,14,16,18,25,34,35,36,37]. Therefore, these postmortem studies suggested that downregulation of Cx43 probably contributes to pathomechanisms of depressive mood or depressive emotional perception [2]. This Cx43 hypothesis was supported by a number of preclinical studies using depressive-like experimental animal models, including chronic unpredictable stress, restraint stress and exogenous corticosterone [6,26,27,38,39,40,41,42]. These distresses, which generate depressive-like behaviors, enhanced astroglial hemichannel permeabilities but suppressed gap-junction permeabilities or Cx43 expression [6,26,27,38,39,40,41,42]. Furthermore, pharmacological studies reported that chronic administrations of several monoamine transporter inhibiting antidepressants, fluoxetine, fluvoxamine and duloxetine increased the mRNA and proteins of Cx43, but paradoxically suppressed hemichannel activities [2,23,26,43,44]. Until recently, the detailed mechanisms of the opposite effects of monoamine transporter inhibiting antidepressants between suppression of hemichannel activity but enhancement of Cx43 expression has remained to be clarified; however, it is speculated that enhancement of homeostatic and prevention of toxic functions due to respective enhanced gap-junction and inhibited hemichannel probably play important roles in the antidepressive action of these monoamine transporter inhibiting antidepressants [3,9]. Indeed, the reduction of the glial population in the dorsolateral prefrontal cortex, orbitofrontal cortex, subgenual cortex anterior cingulate cortex and amygdala of individuals with major depression was reported in numerous studies [45,46,47,48]; however, an increase in glial size was also observed in major depression [49,50]. These abnormalities have not been observed in schizophrenia [51,52].

Contrary to monoamine transporter inhibiting antidepressants, a part of mood-stabilizers enhanced astroglial hemichannel activities. In vitro studies using cortical cultured astrocytes demonstrated that subacute administration of therapeutic-relevant concentration of valproate (VPA), a histone deacetylase inhibiting mood-stabilizing antiepileptic drug [53], increased Cx43 expression in astroglial cytosol fraction without affecting that in the plasma membrane [9]. Typical antipsychotic, haloperidol and mood-stabilizing atypical antipsychotic, clozapine (CLZ), did not affect and increased Cx43 expression in astroglial plasma membrane fraction, respectively [9,44]. Especially, this stimulatory effects of therapeutic-relevant concentration of CLZ on Cx43 protein expression were observed in the plasma membrane fraction, but not in cytosol fraction [9]. Another in vivo study also identified consistent effects of antipsychotics on frontal Cx43 expression, since chronic administration of haloperidol and CLZ decreased and increased frontal Cx43 expression, respectively [43]. Taken together with the previous clinical and preclinical findings, the functional abnormalities of hemichannel activities contribute to pathophysiology of mood disturbance rather than schizophrenia. However, the effects of mood-stabilizing atypical antipsychotics on Cx43 expression and astroglial hemichannel activity has remained to be clarified. Therefore, based on our hypothesis, to explore the mechanisms of effects of mood-stabilizing antipsychotics, the present study determined the concentration-dependent effects of acute and subchronic administrations of CLZ, quetiapine (QTP) and brexpiprazole (BPZ) [54,55,56,57] on astroglial Cx43 expression and l-glutamate release through astroglial hemichannel using cortical primary cultured astrocytes. Additionally, combination of mood-stabilizer, VPA with mood-stabilizing antipsychotics is considered to be the first line medication for the treatment of various phases of bipolar disorder [54,58,59,60,61]. In spite of effectiveness, it has been well known that the rapid dose titration of CLZ during the commencement of CLZ or during VPA administration, increases the risk of CLZ-induced myocarditis/cardiomyopathies [2,3,62]. To clarify the mechanisms of adverse reaction and effectiveness associated with combination therapy of VPA with mood-stabilizing antipsychotics, the present study also determined the acute and subchronic administration of CLZ, QTP and BPZ, after the subchronic administration of therapeutic-relevant concentration of VPA, on astroglial l-glutamate release through activated astroglial hemichannel and Cx43 expression in the plasma membrane using ultra-high-performance liquid-chromatography and capillary immunoblotting system, respectively.

## 2. Results

### 2.1. Effects of Mood-Stabilizing Antipsychotics on Astroglial l-Glutamate Release

Therapeutic-relevant serum concentrations of CLZ, QTP and BPZ are 1~3 μM, 0.3~2.6 μM and 0.03~0.3 μM, respectively [63,64]. Based on these clinical data, in the present study, cortical primary cultured astrocytes were acutely (for 120 min) and subchronically (7 days) administered by therapeutic-relevant and supratherapeutic concentrations of CLZ (1, 3, 10 and 30 μM), QTP (0.3, 1, 3 and 10 μM) and BPZ (0.1, 0.3, 1 and 3 μM). Briefly, to study the effects of concentration-dependent effects of mood-stabilizing antipsychotics on astroglial l-glutamate release, during 21~28 days after culture (DIV), cortical astrocytes were incubated in Dulbecco’s modified Eagle’s medium containing 10% fetal calf serum (fDMEM) containing without (control or acute administration) and with (subchronic administration) antipsychotics for 7 days. At DIV28, after the washout by artificial cerebrospinal fluid (ACSF), cortical primary cultured astrocytes were incubated in ACSF containing antipsychotics for 120 min.

#### 2.1.1. Concentration-Dependent Effects of Acute and Subchronic Administration of Mood-Stabilizing Antipsychotics on Astroglial l-glutamate Release during Resting Stage (Study-1)

**Figure 1:**
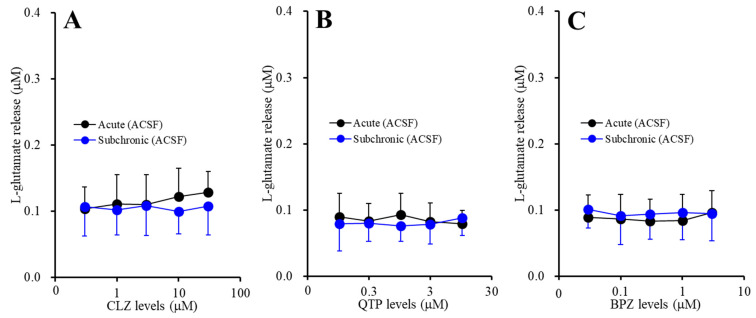
Concentration-dependent effects of the acute (black circles) and subchronic (blue circles) administrations of (**A**) clozapine (CLZ: 1, 3, 10 and 30 μM), (**B**) quetiapine (QTP: 0.3, 1, 3 and 10 μM) and (**C**) brexpiprazole (BPZ: 0.1, 0.3, 1 and 3 μM) on the basal astroglial l-glutamate release. Ordinate: mean ± SD (*n* = 6) of the basal astroglial l-glutamate release (μM), and abscissa: concentration of CLZ, QTP and BPZ (μM).

Neither acute administrations of CLZ (1, 3, 10 and 30 μM), QTP (0.3, 1, 3 and 10 μM) nor BPZ (0.1, 0.3, 1 and 3 μM) affected basal astroglial l-glutamate release (Figure 1A–C). Subchronic administrations of CLZ (1, 3, 10 and 30 μM), QTP (0.3, 1, 3 and 10 μM) and BPZ (0.1, 0.3, 1 and 3 μM) also did not affect basal astroglial l-glutamate release (Figure 1A–C). These results suggest that both acute and subchronic administrations of CLZ, QTP and BPZ do not affect astroglial l-glutamate release during resting stage.

#### 2.1.2. Interaction between Subchronic Administration of Therapeutic-Relevant Concentration of Valproate (VPA) and Acute Administration of Antipsychotics on l-Glutamate Release through Activated Hemichannel (Study-2)

During resting stage, astroglial hemichannel exhibits low opening probability, whereas increased extracellular K^+^ with decreased extracellular Ca^2+^ activates astroglial hemichannel activity [8,9,10,11,12,16,65,66]. Therefore, to study the effects of concentration-dependent effects of mood-stabilizing antipsychotics on astroglial l-glutamate release through activated hemichannel, the cortical cultured astrocytes were stimulated by high (100 mM) K^+^ with Ca^2+^ free ACSF (FCHK-ACSF) for 20 min, according to previous studies [8,9,10,11,12,22,29,67]. To study the interaction between subchronic administrations of therapeutic-relevant concentration of VPA (500 μM), and acute administration of antipsychotics on the astroglial l-glutamate release through activated hemichannel, astrocytes were incubated in fDMEM containing with or without (control) therapeutic-relevant concentration of VPA (500 μM) for 7 days (DIV21~28). At DIV28, after washout, during the pretreatment, the astrocytes were incubated in ACSF containing the same agent with antipsychotics, CLZ (1, 3, 10 and 30 μM), QTP (0.3, 1, 3 and 10 μM) or BPZ (0.1, 0.3, 1 and 3 μM) for 120 min (pretreatment incubation). After pretreatment, the astrocytes were incubated in FCHK-ACSF containing the same agents of pretreatment for 20 min (FCHK-evoked stimulation).

**Figure 2:**
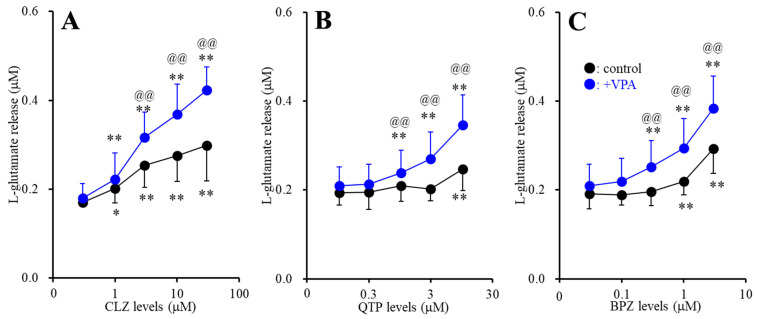
Interaction between subchronic administration of therapeutic-relevant concentration of valproate (VPA: 500 μM) and acute administration of antipsychotics, CLZ (**A**), QTP (**B**) and BPZ (**C**) on l-glutamate release through activated hemichannel. Ordinate: mean ± SD (*n* = 6) of the high (100 mM) K^+^ with Ca^2+^ free artificial cerebrospinal fluid (FCHK-ACSF) evoked astroglial l-glutamate release (μM), and abscissa: concentration of CLZ, QTP and BPZ (μM). Black and blue circles indicate acute antipsychotics administration alone (control) and combination of subchronic VPA with acute antipsychotics administrations (+VPA), respectively. * *p* < 0.05, ** *p* < 0.01: relative to antipsychotics free, @@ *p* < 0.01: relative to control by multivariate analysis of variance (MANOVA) with Tukey’s post hoc test.

Contrary to resting stage, acute administration of both therapeutic-relevant concentration of CLZ (1 and 3 μM) and supratherapeutic concentration of CLZ (10 and 30 μM) for 120 min concentration-dependently increased FCHK-evoked astroglial l-glutamate release through activated hemichannel (Figure 2A). Acute administration of supratherapeutic concentrations of QTP (10 μM) and BPZ (1 and 3 μM) increased FCHK-evoked astroglial l-glutamate release, whereas neither acute administration of therapeutic-relevant concentrations of QTP (0.3 and 1 μM) nor BPZ (0.1 and 0.3 μM) affected FCHK-evoked astroglial l-glutamate release (Figure 2B,C).

Subchronic administration of therapeutic-relevant concentration of VPA (500 μM) enhanced the stimulatory effects of CLZ (F_CLZ_(2.2, 21.9) = 96.5 (*p* < 0.01), F_VPA_(1,10) = 5.0 (*p* < 0.05), F_CLZ*VPA_(2.2,21.9) = 9.4 (*p* < 0.01)), QTP (F_QTP_(4,40) = 64.7(*p* < 0.01), F_VPA_(1,10) = 3.4 (*p* > 0.05), F_QTP*VPA_(4,40) = 14.4 (*p* < 0.01)) and BPZ (F_BPZ_(4, 40) = 122.4 (*p* < 0.01), F_VPA_(1,10) = 3.8 (*p* > 0.05), F_BPZ*VPA_(4,40) = 8.5 (*p* < 0.01)) (Figure 2A–C). Indeed, after the subchronic administration of VPA, therapeutic-relevant concentration of CLZ, QTP and BPZ increased FCHK-evoked astroglial l-glutamate release through activated hemichannel (Figure 2A–C).

#### 2.1.3. Interaction between Subchronic Administrations of Therapeutic-Relevant Concentration of VPA and Antipsychotics on l-Glutamate Release through Activated Hemichannel (Study-3)

To study the interaction between subchronic administrations of therapeutic relevant concentration of VPA (500 μM) and antipsychotics, CLZ (1, 3, 10 and 30 μM), QTP (0.3, 1, 3 and 10 μM) or BPZ (0.1, 0.3, 1 and 3 μM) for 7 days on the astroglial l-glutamate release through activated hemichannel, during DIV21 and DIV28, astrocytes were incubated in fDMEM containing antipsychotics with or without (control) therapeutic-relevant concentration of VPA (500 μM) for 7 days. At DIV28, after washout, during the pretreatment, the astrocytes were incubated in ACSF containing the same agents for 120 min (pretreatment incubation). After pretreatment, the astrocytes were incubated in FCHK-ACSF containing the same agents of pretreatment for 20 min.

**Figure 3:**
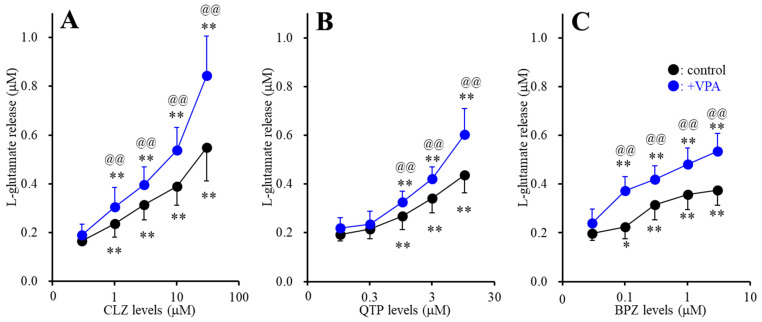
Interaction between subchronic (for 7 days) administration of therapeutic-relevant concentration of VPA (500 μM) and antipsychotics, CLZ (**A**), QTP (**B**) and BPZ (**C**) on l-glutamate release through activated hemichannel. Ordinate: mean ± SD (*n* = 6) of the FCHK-evoked astroglial l-glutamate release (μM), and abscissa: concentration of CLZ, QTP and BPZ (μM). Black and blue circles indicate acute antipsychotics administration alone (control) and combination of subchronic administration of VPA with antipsychotics (+VPA), respectively. * *p* < 0.05, ** *p* < 0.01: relative to antipsychotics free, @@ *p* < 0.01: relative to control by MANOVA with Tukey’s post hoc test.

Subchronic administration of CLZ, QTP and BPZ concentration-dependently increased FCHK-evoked astroglial l-glutamate release through activated hemichannel (Figure 3A–C). Especially, subchronic administration of both therapeutic-relevant concentration of CLZ (1 and 3 μM), QTP (1 μM) and BPZ (0.1 and 0.3 μM) for 7 days increased FCHK-evoked astroglial l-glutamate release (Figure 3A–C). Subchronic administration of therapeutic-relevant concentration of VPA (500 μM) enhanced the stimulatory effects of CLZ (F_CLZ_(1.7, 16.7) = 161.1 (*p* < 0.01), F_VPA_(1,10) = 8.3 (*p* < 0.05), F_CLZ*VPA_(1.7,16.7) = 11.2 (*p* < 0.01)), QTP (F_QTP_(2.1,20.5) = 228.8 (*p* < 0.01), F_VPA_(1,10) = 5.2 (*p* < 0.05), F_QTP*VPA_(2.1,20.5) = 12.2 (*p* < 0.01)) and BPZ (F_BPZ_(3.0,29.8) = 257.0 (*p* < 0.01), F_VPA_(1,10) = 13.2 (*p* < 0.01), F_BPZ*VPA_(3.0,29.8) = 17.8 (*p* < 0.01)) (Figure 3A–C).

#### 2.1.4. Effects of Cx43 Inhibitor on Astroglial l-glutamate Release through Activated Hemichannel Enhanced by Acute Administration of Antipsychotics (Study-4)

To clarify the mechanisms of stimulatory effects of antipsychotics on FCHK-evoked astroglial l-glutamate release, at DIV28 after washout, during the pretreatment, the astrocytes were incubated in ACSF containing CLZ (30 μM), QTP (10 μM) or BPZ (3 μM) with or without selective Cx43 inhibitor, N-terminal transactivator of transcription GAP19 (TAT-GAP19: 10 μM) for 120 min [8]. After pretreatment, the astrocytes were incubated in ACSF (basal release) or FCHK-ACSF (FCHK-evoked release) containing the same agents of pretreatment for 20 min.

**Figure 4:**
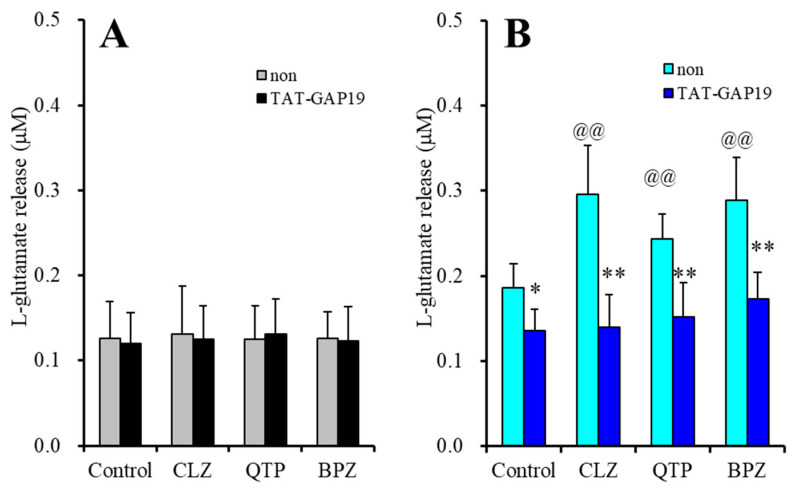
Effects of selective Cx43 inhibitor, N-terminal transactivator of transcription GAP19 (TAT-GAP19) on stimulatory effects of antipsychotics, CLZ (30 μM), QTP (10 μM) and BPZ (3 μM), on basal (**A**) and FCHK-evoked (**B**) astroglial l-glutamate releases. Ordinate: mean ± SD (*n* = 6) of astroglial l-glutamate release (μM). Light and dark color columns indicate the incubation without (non) and with TAT-GAP19, respectively. * *p* < 0.05, ** *p* < 0.01: relative to non, @@ *p* < 0.01 relative to control by MANOVA with Tukey’s post-hoc test.

Neither antipsychotics nor TAT-GAP19 affected basal astroglial l-glutamate release (Figure 4A). TAT-GAP19 (10 μM) inhibited FCHK-evoked l-glutamate release (Figure 4B). TAT-GAP19 also prevented the stimulatory effects of 30 μM CLZ (F_CLZ_(1,20) = 41.4 (*p* < 0.01), F_GAP19_(1,20) = 12.6 (*p* < 0.01), F_CLZ*GAP19_(1,20) = 11.1 (*p* < 0.01)), 10 μM QTP (F_QTP_(1,20) = 31.0 (*p* < 0.01), F_GAP19_(1,20) = 8.5 (*p* < 0.01), F_QTP*GAP19_(1,20) = 4.7 (*p* < 0.05)) and 3 μM BPZ (F_BPZ_(1,20) = 33.3 (*p* < 0.01), F_GAP19_(1,20) = 24.0 (*p* < 0.05), F_BPZ*GAP19_(1,20) = 5.3 (*p* < 0.05)) (Figure 4B).

These results indicated that astroglial hemichannel could not contribute to astroglial basal l-glutamate release during resting stage, whereas FCHK-evoked l-glutamate release is composed of its release through activated Cx43 containing hemichannel, since selective Cx43 inhibitor, TAT-GAP19 prevented the FCHK-evoked l-glutamate release. Therefore, the stimulatory effects of 30 μM CLZ, 10 μM QTP and 3 μM BPZ acutely enhanced the function of activated astroglial hemichannel resulting in increasing astroglial l-glutamate release through activated Cx43 containing hemichannel.

#### 2.1.5. Effects of Acute Administration of Protein Kinase B (Akt) Inhibitor on Astroglial l-glutamate Release through Activated Hemichannel Enhanced by Acute and Subchronic Administration of Antipsychotics, after the Subchronic Administration of Therapeutic-Relevant Concentration of VPA (Study-5)

To clarify the mechanisms of stimulatory effects of antipsychotics on FCHK-evoked astroglial l-glutamate release, during DIV21~28, astrocytes were incubated in the fDMEM containing with (subchronic administration) or without (control or acute administration) antipsychotics, CLZ (30μM), QTP (10 μM) or BPZ (3 μM). At DIV28, astrocytes were incubated in fDMEM containing with or without protein kinase B (Akt) inhibitor, 10-[4′-(N,N-diethylamino)butyl]-2-chlorophenoxazine hydrochloride (DEBC: 10 μM) for 120 min (pre-incubation). After washout, during the pretreatment, the astrocytes were incubated in FCHK-ACSF containing the same agent of pre-incubation for 20 min.

**Figure 5:**
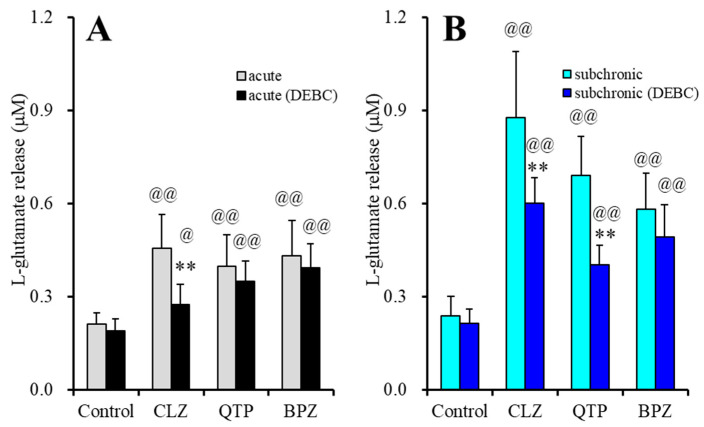
Effects of acute exposure to Akt inhibitor, 10-[4′-(N,N-diethylamino)butyl]-2-chlorophenoxazine hydrochloride (DEBC: 10 μM) for 120 min on enhanced l-glutamate release through activated hemichannel induced by combination of subchronic administration of therapeutic-relevant concentration of VPA (500 μM) with acute (**A**) and subchronic (**B**) administration of CLZ (30 μM), QTP (10 μM) and BPZ (3 μM). Ordinate: mean ± SD (*n* = 6) of FCHK-evoked astroglial l-glutamate release (μM). Light and dark color columns indicate the incubation without (non) and with DEBC (10 μM), respectively. ** *p* < 0.01: relative to non, @ *p* < 0.05, @@ *p* < 0.01 relative to control by MANOVA with Tukey’s post-hoc test.

Acute administration of Akt inhibitor, 10 μM DEBC, for 2 hr did not affect FCHK-evoked l-glutamate release (Figure 5A,B). After the subchronic administration of therapeutic-relevant concentration of VPA, acute administration of CLZ (30 μM), QTP (10 μM) and BPZ (3 μM) increased FCHK-evoked l-glutamate release (Figure 5A). DEBC (10 μM) suppressed the stimulatory effect of acute administration of CLZ on FCHK-evoked astroglial l-glutamate release (F_CLZ_(1,20) = 33.3 (*p* < 0.01), F_DEBC_(1,20) = 12.8 (*p* < 0.01), F_CLZ*DEBC_(1,20) = 7.8 (*p* < 0.01)), but did not affect those of acute administration of QTP (F_QTP_(1,20) = 41.9 (*p* < 0.01), F_DEBC_(1,20) = 1.8 (*p* > 0.1), F_QTP*DEBC_(1,20) = 0.3 (*p* > 0.1)) or BPZ (F_BPZ_(1,20) = 49.6 (*p* < 0.01), F_DEBC_(1,20) = 1.0 (*p* > 0.1), F_BPZ*DEBC_(1,20) = 0.1 (*p* > 0.1)) (Figure 5A).

Subchronic administration of therapeutic-relevant concentration of VPA with CLZ (30 μM), QTP (10 μM) or BPZ (3 μM) increased FCHK-evoked l-glutamate release (Figure 5B). DEBC (10 μM) suppressed the stimulatory effect of subchronic administration of CLZ (F_CLZ_(1,20) = 108.5 (*p* < 0.01), F_DEBC_(1,20) = 9.3 (*p* < 0.01), F_CLZ*DEBC_(1,20) = 6.6 (*p* < 0.01)) and QTP (F_QTP_(1,20) = 95.6 (*p* < 0.01), F_DEBC_(1,20) = 22.6 (*p* < 0.01), F_QTP*DEBC_(1,20) = 16.2 (*p* < 0.01)), but did not affect that of BPZ (F_BPZ_(1,20) = 77.5 (*p* < 0.01), F_DEBC_(1,20) = 2.5 (*p* > 0.1), F_BPZ*DEBC_(1,20) = 0.8 (*p* > 0.1)) (Figure 5B).

These results suggest that the distinct mechanisms of stimulatory effects of CLZ, QTP and BPZ on astroglial l-glutamate release through activated hemichannels. Both CLZ and QTP enhance astroglial l-glutamate release through activated hemichannel via activation of Akt-dependent process; however, the onset of activation of Akt-dependent process induced by CLZ is within hours order, but that by QTP requires longer than days order. Contrary to CLZ or QTP, BPZ enhances astroglial l-glutamate release through activated hemichannel, but the stimulation by BPZ is independent on Akt signaling.

### 2.2. Effects of Mood-Stabilizing Antipsychotics on Expression of Cx43 Protein in the Astroglial Plasma Membrane Fraction

It has been well known that VPA enhances transcription of a number of mRNA via histone deacetylase inhibition [9,53,68,69]. We have already demonstrated that subchronic administration of therapeutic-relevant concentration of VPA increased Cx43 expression in the astroglial cytosol fraction without affecting that in the astroglial plasma membrane fraction [9]. Furthermore, neither acute nor subchronic administration of therapeutic-relevant concentration of CLZ weakly affected Cx43 protein expression in the plasma membrane, whereas combination of subchronic administration of therapeutic-relevant concentration of VPA with CLZ drastically increased Cx43 expression in the plasma membrane fraction [9]. Other line study reported that trafficking of Cx43 to the plasma membrane is regulated by several intracellular phosphorylation signaling, including Akt [2,3,10,22]. Based on the previous findings, therefore, to explore the effects of therapeutic-relevant concentration of CLZ (3 μM), QTP (1 μM) and BPZ (0.3 μM) on Cx43 expression in the plasma membrane fraction, the interaction among antipsychotics, VPA and Akt inhibitor (DEBC) on Cx43 expression in the astroglial plasma membrane fraction using capillary immunoblotting system.

#### 2.2.1. Effects of Subchronic Administration of Therapeutic-Relevant Concentrations of Antipsychotics alone on Cx43 Expression in the Plasma Membrane Fraction

To clarify the subchronic administration of therapeutic-relevant concentration of antipsychotics on Cx43 expression in the plasma membrane, astrocytes were incubated in the fDMEM containing CLZ (3 μM), QTP (1 μM) or BPZ (0.3 μM) for 7 days (from DIV21 to DIV 28). Three antipsychotics subchronically did not affect Cx43 expression in the plasma membrane fractions during resting stage. Similar to the present results, in our previous study, subchronic administration of supratherapeutic concentration of CLZ (30 μM) increased Cx43 expression in the astroglial plasma membrane, but therapeutic-relevant concentration of CLZ (3 μM) did not affect.

#### 2.2.2. Effects of Acute and Subchronic Administrations of Therapeutic-Relevant Concentration of Antipsychotics on Cx43 Expression in the Astroglial Plasma Membrane, after Subchronic Administration of Therapeutic-Relevant Concentration of VPA

**Figure 6:**
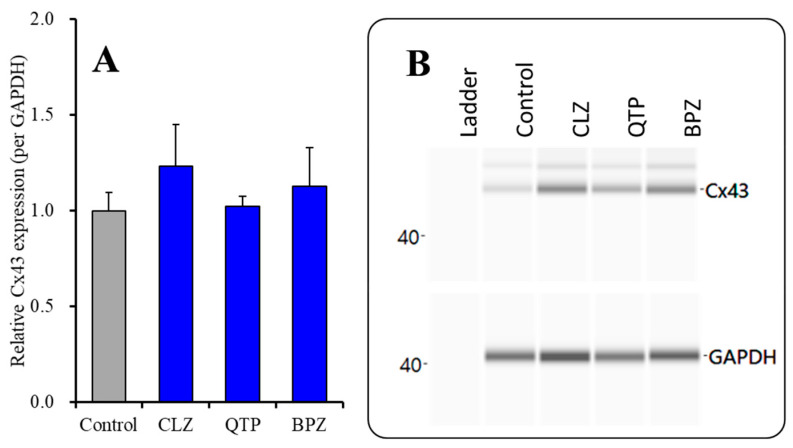
Effects of subchronic administration of therapeutic-relevant concentration of antipsychotics, CLZ (3 μM), QTP (1 μM) and BPZ (0.3 μM), on Cx43 protein expression in the plasma fraction (**A**) and their pseudo-gel images, using capillary immunoblotting (**B**). Ordinate: mean ± SD (*n* = 6) of the relative protein level of Cx43 (per GAPDH).

**Figure 7:**
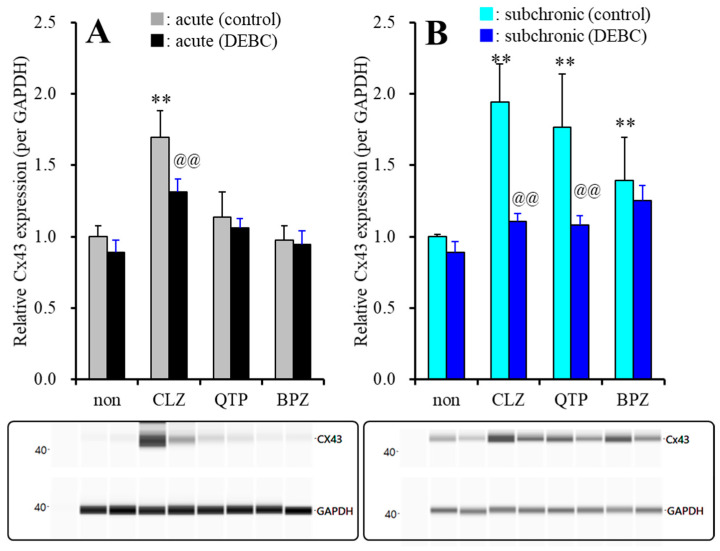
Interaction between acute administration of therapeutic-relevant concentration of antipsychotics and Akt inhibitor (DEBC) on Cx43 protein expression in the astroglial plasma membrane fraction, after subchronic administration of therapeutic-relevant concentration of VPA (500 μM) (**A**). Interaction between subchronic administration of therapeutic-relevant concentration of antipsychotics and DEBC on Cx43 protein expression in the astroglial plasma membrane fraction, after subchronic administration of therapeutic-relevant concentration of VPA (**B**). Lower panels indicate their pseudo-gel images, using capillary immunoblotting. Ordinate: mean ± SD (*n* = 6) of the relative protein level of Cx43 (per GAPDH). Effects of antipsychotics and Akt inhibitor (DEBC: 10 μM) on Cx43 expression in the plasma membrane fraction of the primary cultured astrocytes were analyzed by MANOVA with Tukey’s post hoc test (** *p* < 0.01 vs. non, @@ *p* < 0.01 vs. control).

Subchronic administration of therapeutic-relevant concentrations of CLZ (3 μM for 7 days) alone did not affect Cx43 expression in the plasma membrane (Figure 6), whereas after subchronic administration of therapeutic-relevant concentration of VPA (500 μM) for 7 days (during DIV21~28), acute administration of CLZ (3 μM for 120 min) increased astroglial Cx43 expression in the plasma membrane fraction (Figure 7A). Acute exposure to Akt inhibitor, 10 μM DEBC (for 120 min) suppressed the CLZ-induced elevation of Cx43 expression in the plasma membrane fraction (F_CLZ_(1,20) = 134.5 (*p* < 0.01), F_DEBC_(1,20) = 26.2 (*p* < 0.01), F_CLZ*DEBC_(1,20) = 8.1 (*p* < 0.05)) (Figure 7A). Contrary to CLZ, after subchronic administrations of VPA (500 μM), neither acute administration of therapeutic-relevant concentration of QTP (1 μM) nor BPZ (0.3 μM) affected Cx43 expression in the plasma membrane fraction (Figure 7A). Therefore, CLZ acutely enhances Cx43 trafficking to the plasma membrane via activation of Akt signaling, whereas neither QTP nor BPZ acutely affect Akt signaling.

Similar to acute CLZ administration, subchronic administration of therapeutic-relevant concentration of VPA (500 μM) with CLZ (3 μM) for 7 days also increased Cx43 expression in the plasma membrane fraction (Figure 7B). The increased Cx43 expression in the plasma membrane fraction induced by CLZ was also suppressed by DEBC (10 μM) (F_CLZ_ (1,20) = 98.2 (*p* < 0.01), F_DEBC_(1,20) = 65.6 (*p* < 0.01), F_CLZ*DEBC_(1,20) = 38.8 (*p* < 0.01)) (Figure 7B). Subchronic administration of therapeutic-relevant concentration of QTP (1 μM) and BPZ (0.3 μM) also increased Cx43 expression in the plasma membrane (Figure 7B). The increased Cx43 expression induced by QTP was suppressed by DEBC (10 μM) (F_QTP_(1,20) = 37.3 (*p* < 0.01), F_DEBC_(1,20) = 25.7 (*p* < 0.01), F_QTP*DEBC_(1,20) = 13.5 (*p* < 0.05)), but that induced by BPZ was not inhibited by DEBC (10 μM) (F_BPZ_(1,20) = 31.2 (*p* < 0.05), F_DEBC_(1,20) = 3.4 (*p* > 0.05), F_BPZ*DEBC_(1,20) = 0.1 (*p* > 0.05)) (Figure 7B). Thus, both CLZ and QTP activate Cx43 trafficking to plasma membrane via possibly enhancement of Akt signaling, but the Akt signaling activation induced by CLZ (within hours range) is rapid onset compared to QTP (within days range). Contrary to CLZ and QTP, subchronic administration of therapeutic-relevant concentration of BPZ Cx43 trafficking to the plasma membrane, but the BPZ induced Cx43 trafficking is not mediated by Akt signaling.

## 3. Discussion

Based on both clinical and preclinical findings, several reports suggest that tripartite synaptic transmission contributes to pathophysiology of mood disorders [2,3]. Indeed, postmortem studies revealed that expressions of mRNA or protein of Cx43 were downregulated in the locus coeruleus, frontal cortex and thalamus of patients with mood disorders [2,30,31,32,33]. Additionally, monoamine transporter inhibiting antidepressants enhance transcription of Cx43 but suppress the hemichannel permeability [2,23,26,43,44]. Contrary to monoamine transporter inhibiting antidepressants, the effects of mood-stabilizing antipsychotics on astroglial transmission associated with Cx43 have remained to be clarified [9,43,44]. To explore the novel mood-stabilizing mechanisms, the present study determined the effects of mood-stabilizing antipsychotics, CLZ, QTP and BPZ [54,55,56,57] on Cx43 expression and Cx43 containing hemichannel using primary cultured rat cortical astrocytes. The results in this study, are summarized in Table 1.

### 3.1. Mechanisms of Mood-Stabilizing Antipsychotics, CLZ, QTP and BPZ, on Astroglial l-Glutamate Release through Hemichannel

In the present study, neither acute nor subchronic administrations of CLZ, QTP and BPZ affected basal astroglial l-glutamate release. Therefore, these three mood-stabilizing antipsychotics do not affect (activate) directly hemichannel permeabilities, since selective Cx43 hemichannel inhibitor, TAT-GAP19 could not affect basal astroglial l-glutamate release. Contrary to basal release, three mood-stabilizing antipsychotics enhanced astroglial l-glutamate release through activated Cx43 containing hemichannel, since the stimulatory effects of CLZ, QTP and BPZ on FCHK-evoked astroglial l-glutamate release were suppressed by TAT-GAP19. Interestingly, both acute and subchronic administrations of therapeutic-relevant concentration of CLZ enhanced astroglial l-glutamate release through activated astroglial hemichannel; however, therapeutic-relevant concentrations of QTP and BPZ acutely did not affect but subchronically enhanced astroglial l-glutamate release through activated Cx43 containing hemichannel (supratherapeutic concentrations of QTP and BPZ acute increased FCHK-evoked l-glutamate release). Therefore, these time-dependent discrepancy (acute vs. subchronic administrations of therapeutic-relevant concentration) between QTP, BPZ and CLZ on astroglial l-glutamate release through activated hemichannel suggests that the stimulatory effects of QTP and BPZ on astroglial Cx43 containing hemichannel activities requires higher concentration acutely and/or time-dependent processes compared to CLZ. In other words, these three mood-stabilizing antipsychotics enhance astroglial l-glutamate release through activated Cx43 hemichannel, whereas the stimulatory mechanisms between therapeutic-relevant concentration of CLZ, QTP and BPZ on astroglial l-glutamate release are possibly different.

To clarify the mechanisms of CLZ, QTP and BPZ on astroglial transmission associated with Cx43, time-dependent (acute and subchronic administrations) effects of CLZ, QTP and BPZ on expression of Cx43 in the astroglial plasma membrane were determined. During resting stage, neither subchronic administration of therapeutic-relevant concentration of CLZ, QTP nor BPZ affected Cx43 expression in the plasma membrane, whereas after the subchronic administration of therapeutic-relevant concentration of VPA (500 μM), acute administration of therapeutic-relevant concentration of CLZ (3 μM) increased Cx43 expression in the plasma membrane fraction, but those of QTP and BPZ did not affect. Subchronic administration of therapeutic-relevant concentration of VPA increased Cx43 expression in cytosol without affecting that in the plasma membrane due to enhancement of transcription process via histone deacetylase inhibition [9,53]. Contrary to acute administration, subchronic administration of therapeutic-relevant concentration of VPA with CLZ, QTP and BPZ increased Cx43 expression in the plasma membrane. Therefore, these three mood-stabilizing antipsychotics, CLZ, QTP and BPZ enhance the trafficking of Cx43 to astroglial plasma membrane, whereas the time-dependency of the promoting trafficking Cx43 to plasma membrane of CLZ is rapid onset rather than those of QTP and BPZ. Turnover of Cx43 is regulated by both transcription and post-transcription processes. The transcription process is regulated by various factors, activator protein 1 complex (Sp1), cyclic adenosine monophosphate (cAMP), wingless (Wnt) pathway and epigenetic factor (histone modifications, DNA methylation and microRNA species) [2,70]. The post-transcription process is regulated by the phosphorylation, acetylation, nitrosylation, sumoylation and ubiquitylation factors, including phosphorylation of Akt [2,10,22,70]. Previous study revealed that CLZ enhanced the post-transcription process rather than transcription process associated with Cx43 turnover [2,3,9]. We have already proposed the candidate hypothesis that various clinical and adverse effects of CLZ is possibly mediated by Akt function [2,3,9]. Based on our hypothesis, the present study determined the interaction between mood-stabilizing antipsychotics (CLZ, QTP and BPZ) and Akt inhibitor, DEBC on astroglial l-glutamate release and Cx43 expression in the plasma membrane. According to our expectation, the stimulatory effects of CLZ and QTP on Cx43 expression in the plasma membrane fraction and astroglial l-glutamate release through activated hemichannel were inhibited by Akt inhibitor (DEBC), whereas those of BPZ was Akt insensitive. Therefore, therapeutic-relevant concentration of CLZ, QTP and BPZ commonly enhance astroglial l-glutamate release through activated Cx43 hemichannel due to increased functional Cx43 expression in the astroglial plasma membrane, but the mechanisms of upregulation of Cx43 induced by three antipsychotics were not identical. Both CLZ and QTP enhance Cx43 trafficking to the plasma membrane via Akt signaling, but the onset of Akt signaling activation induced by CLZ (hours order) is rapid rather than that by QTP (days order). Contrary to CLZ and QTP, BPZ also enhances Cx43 trafficking but this stimulatory effects of BPZ on Cx43 trafficking is probably mediated by Akt independent signaling.

### 3.2. Mechanisms of Clinical Action of Mood-Stabilizing Antipsychotics Associated with Cx43

In spite of no evidence indicating any abnormalities of Cx43 in genomes of individuals with schizophrenia or bipolar disorder, the accumulating findings suggest that the functional abnormalities of hemichannels lead to severe cognitive dysfunction in schizophrenia via disorganization in neuro-glial networks and transmission dysfunction in specific regions [2,22,71,72]. Therefore, the dysfunction of hemichannel plays important roles in the pathophysiology but not pathogenesis of schizophrenia or bipolar disorder as a possible reversible functional abnormality that is able to be compensated by psychopharmacological medication. Postmortem studies revealed that expression of mRNA and protein of Cx43 were decreased in several brain regions of individuals with major depression or suicide [2]. Similarly, preclinical studies also indicated that downregulation of Cx43 was also observed in the brain of several depression rodent models [2]. Monoamine transporter inhibiting antidepressants increased Cx43 synthesis but suppressed astroglial hemichannel activity, whereas non-selective inhibitor, carbenoxolone (suppression of functions of both gap-junction and hemichannel) and zonisamide (suppression of Cx43 synthesis and hemichannel activity) generate depressive mood and anhedonia [2,8,10,22,26]. These clinical and preclinical findings suggest that either attenuation of Cx43 synthesis or inhibition of gap-junction function contributes to depressive mood or dysfunction of emotional cognition/perception [2,6,22]. A meta-analysis and systematic review study reported that CLZ, QTP and BPZ significantly improves various symptoms of schizophrenia, including positive and negative symptoms [73]. CLZ is established the sole approval antipsychotic agent for the treatment of antipsychotics-resistant schizophrenia and one of the most effective antipsychotics [74]. Another meta-analysis study reported that CLZ, BPZ and QTP therapies for the management of acute phase of bipolar disorder and bipolar depression were efficacious [54,55,56,57,75,76]. Taken together with clinical findings, the present results suggest the candidate mechanisms that enhanced functional astroglial Cx43 is, at least partially, involved in the mood-stabilizing antipsychotic actions of CLZ, QTP and BPZ. The opposite effects of mood-stabilizing antipsychotics and monoamine transporter inhibiting antidepressants on astroglial hemichannel activity suggest that enhancement and suppression of astroglial Cx43 containing hemichannel activity probably contribute to anti-manic/mood-stabilizing and anti-depressive actions, respectively. However, the present study did not clarify the effects of monoamine transporter inhibiting antidepressants or mood-stabilizing antipsychotics on astroglial gap-junction activities. Therefore, to clarify the detailed pathophysiological contribution of astroglial Cx43, we shall report the effects of mood-stabilizing antipsychotics and monoamine transporter inhibiting antidepressants on function of Cx43 containing astroglial gap-junction in the future.

It has been well established the higher vigilance for seizure is warranted during treatment with CLZ and QTP [77,78]. Recently, preclinical findings using several epileptic/convulsive animal models have displayed the possibility that upregulation/hyperfunction of Cx43 contributes to development of epileptogenesis/ictogenesis [2,10,11,12,22,79,80,81,82]. Furthermore, pharmacodynamic studies reported that Cx43 is one of the major targets of several anti-seizure agents, zonisamide, lacosamide, brivaracetam and VPA [8,9,11,12,67]. Astroglial hemichannel is activated by depolarization and exposure to transient toxic (100 mM) or persistent pathological (10 mM) extracellular K^+^, and active state of hemichannel continues several hours [8,9,10,11,12,22,67,79]. Therefore, the stimulatory effects of CLZ and QTP on astroglial Cx43 containing hemichannel activity seem to be rational mechanisms regarding the adverse seizure reaction induced by CLZ and QTP. Contrary to CLZ and QTP, although the seizure-inducing effect of BPZ has not been clarified in clinical or preclinical studies (there are no reports of the BPZ-induced seizure); however, the present demonstration suggests that long-term exposure to therapeutic-relevant concentration of BPZ possibly shifts to enhancement of seizure susceptibility via astroglial Cx43 upregulation in the plasma membrane. Indeed, generally, aripiprazole, which is similar structural derivate and pharmacodynamic profile with BPZ [18,83], is considered to be well tolerated; however, aripiprazole is independently associated with greater seizure risk which enhanced further with an increase in the number of other antipsychotics, including QTP [84,85]. It should be notable the accumulating clinical experience regarding BPZ-induced seizure reaction in the future.

CLZ and QTP were associated with diabetes and weight gain, whereas BPZ was associated with less weight gain rather than QTP [73]. Considering with these previous clinical findings, the activation of astroglial l-glutamate release through Cx43 upregulation containing hemichannel induced by CLZ, QTP and BPZ probably plays important roles in the pathophysiology of mood-stabilizing antipsychotics, but cannot directly/fundamentally provide the adverse reaction regarding antipsychotics-induced seizure and glucose intolerability. Phosphorylated Akt leads to trafficking glucose transporter to the plasma membrane [86] and suppresses glucose glycogen synthase kinase 3 resulting in the enhancement of glycogen synthesis [87]. Therefore, the stimulatory effects of CLZ and QTP on Akt signaling contribute to the weight gain, but seem to provide the opposite action on CLZ- and QTP-induced glucose intolerance; however, upregulated Akt signaling, which was observed in the models of insulin resistance, generated insulin desensitization [88]. Indeed, CLZ attenuated the early events of insulin signaling, inhibiting insulin receptor tyrosine auto-phosphorylation and kinase activity and insulin-stimulated Akt phosphorylation [89]. Therefore, taken together with these previous findings, long-term treatment or rapid titration of CLZ probably leads to persistent up-regulation of Akt resulting in the inhibition of insulin signaling. According to this our hypothesis, the different risks of glucose intolerance and weight gain between QTP and BPZ might be also explained by upregulation of Akt signaling.

Similar to CLZ and QTP, the serotonin receptor binding profile of BPZ displays the feature of atypical antipsychotic agent, since BPZ is a potent partial agonist of serotonin 5-HT1A receptor and serotonin 5-HT2A receptors antagonism [1,5], but BPZ is the dopamine D2 receptor partial agonist [90,91]. Activation of dopamine D2 receptor suppresses Akt signaling leading to disinhibition of glucose glycogen synthase kinase 3 [92,93]. Considering with the partial agonistic profile of BPZ to dopamine D2 receptor, the lesser activation of Akt activity induced by therapeutic-concentration of BPZ compared to CLZ and QTP might be modulated by the partial agonistic action of BPZ; however, the intrinsic activity of BPZ at dopamine D2 receptors is lower than 20% [91]. Therefore, we shall determine the concentration-dependent effects of CLZ, QTP and BPZ on Akt activity. To explore the more detailed mechanisms of these three mood-stabilizing atypical antipsychotics on tripartite synaptic transmission, we shall study the effects of CLZ, QTP and BPZ on various transmissions including monoaminergic transmission in vivo.

## 4. Materials and Methods

### 4.1. Chemical Agents

Clozapine (CLZ) and sodium valproate (VPA) were obtained from Fujifilm-Wako (Osaka, Japan). Brexpiprazole (BPZ), quetiapine fumarate (QTP), selective Cx43 inhibitor, TAT-conjugated Gap19 (GAP19) and 10-[4-(N,N-diethylamino)butyl]-2-chlorophenoxazine hydrochloride (DEBC: Akt inhibitor) were obtained from Funakoshi (Tokyo, Japan). All compounds were prepared on the day of the experiment. TAT-GAP19, VPA and DEBC were dissolved in ACSF or fDMEM directly. CLZ was initially dissolved in 50 mM with 1N HCl, then diluted to 1 mM with ACSF or fDMEM. BPZ and QTP were initially dissolved in dimethyl sulfoxide at 50 mM. The final dimethyl sulfoxide concentration was lower than 0.1% (*v*/*v*).

### 4.2. Preparation of Primary Astrocyte Culture

All animal care and experimental procedures described in this report complied with the Ethical Guidelines established by the Institutional Animal Care and Use Committee at Mie University, Japan (No. 2019-3-R2, 24 May 2019) and are reported in accordance with the Animal Research: Reporting of In Vivo Experiments (ARRIVE) guidelines. Astrocytes were prepared using a protocol adapted from previously described methods [8,10,13,17,20,25].

Pregnant Sprague-Dawley rats (SLC, Sizuoka, Japan) were housed individually in cages and kept in air-conditioned rooms (temperature, 22 ± 2 °C) set at 12 h light/dark cycle, with free access to food and water. Cultured astrocytes were prepared from cortical astrocyte cultures of neonatal Sprague-Dawley rats (N = 54) sacrificed by decapitation at 0–24 h of age. The cerebral hemispheres were removed under dissecting microscope. Tissue was chopped into fine pieces using scissors and then triturated briefly with micropipette. Suspension was filtered using 70 µm nylon mesh (BD, Franklin Lakes, NJ, USA) and centrifuged. Pellets were then resuspended in 10 mL Dulbecco’s modified Eagle’s medium containing 10% foetal calf serum (fDMEM), which was repeated three times. After culture for 14 days (DIV14), contaminating cells were removed by shaking in standard incubator for 16 h at 200 rpm. On DIV21, astrocytes were removed from flasks by trypsinization and seeded directly onto translucent poly ethylene terephthalate (PET) membrane (1.0 μm) with 24-well plates (BD) at a density of 100 cells/cm^2^ for experiments from DIV21 to DIV28, the culture medium (fDMEM) was changed twice a week, and CLZ (1, 3, 10 and 30 μM), QTP (0.3, 1, 3 and 10 μM), BPZ (0.1, 0.3, 1 and 3 μM) or VPA (500 μM) were added for subchronic administrations (7 days). On DIV28, cultured astrocytes were washed out using ACSF, and this was repeated three times.

The ACSF comprised NaCl 150.0 mM, KCl 3.0 mM, CaCl_2_ 1.4 mM, MgCl_2_ 0.8 mM and glucose 5.5 mM, buffered to pH 7.3 with 20 mM HEPES buffer. After the washout, astrocytes were incubated in ACSF (100 μL translucent PET membrane) containing CLZ (1, 3, 10 and 30 μM), QTP (0.3, 1, 3 and 10 μM), BPZ (0.1, 0.3, 1 and 3 μM) or VPA (500 μM) at 35 °C for 60 min in CO_2_ incubator (pretreatment incubation). After the pretreatment, astrocytes were then incubated in ACSF, 100 mM K^+^ with Ca^2+^ free (FCHK-ACSF) containing the same agents of pretreatment (20 min) and collection of the ACSF or FCHK-ACSF for analysis. Each 100 μL of collected ACSF or FCHK-ACSF was filtered by Vivaspin 500-3K (Sartorius, Goerringen, Germany) and freeze-dried for storage at −80 °C until needed for analyses. The composition of NaCl and KCl in fDMEM and ACSF were modified to maintain isotonicity and ionic strength [10,11,12,13,20,21].

After the sampling of astroglial transmitter releases, plasma membrane proteins of cultured astrocytes were extracted using Minute Plasma Membrane Protein Isolation Kit (Invent Biotechnologies, Plymouth, MN, USA). Plasma membrane fractions were solubilized by radio immunoprecipitation assay buffer (Fujifilm-Wako, Osaka, Japan) containing protease inhibitor cocktail (Nacalai Tesque, Kyoto, Japan) [7,67].

### 4.3. Ultra-High-Performance Liquid Chromatography (UHPLC)

l-glutamate levels were determined by using UHPLC equipped with xLC3185PU (Jasco, Tokyo, Japan) and fluorescence detection (xLC3120FP, Jasco) following dual derivatization with isobutyryl-l-cysteine/o-phthalaldehyde. The derivatized samples (5 μL aliquots) were injected via an autosampler (xLC3059AS, Jasco). The analytical column (YMC Triat C18, particle 1.8 μm, 50 × 2.1 mm, YMC, Kyoto, Japan) was maintained at 45 °C, and the flow rate was set to 500 μL/min. A linear gradient elution program was used over a period of 10 min with mobile phases A (0.05 M citrate buffer, pH 5.0) and B (0.05 M citrate buffer containing 30% acetonitrile and 30% methanol, pH 3.5). The excitation/emission wavelengths of the fluorescence detector were set to 280/455 nm [15,29,34,35,79].

### 4.4. Capillary Immunoblotting Analysis

The capillary immunoblotting analysis was performed, using Wes (ProteinSimple, Santa Clara, CA, USA), according to the ProteinSimple user manual. The lysates of the primary cultured astrocytes were mixed with a master mix (ProteinSimple) to a final concentration of 1 × sample buffer, 1 × fluorescent molecular weight marker and 40 mM dithiothreitol and then heated at 95 °C for 5 min. The samples, blocking reagents, primary antibodies, HRP-conjugated secondary antibodies, chemiluminescent substrate (SuperSignal West Femto: Thermo Fisher Scientific, Waltham, MA, USA), and separation and stacking matrices were also dispensed to the designated wells in a 25 well plate. After plate loading, the separation electrophoresis and immunodetection steps took place in the capillary system and were fully automated. A capillary immunoblotting analysis was carried out at room temperature, and the instrument’s default settings were used. Capillaries were first filled with a separation matrix followed by a stacking matrix, with about 40 nL of the sample used for loading. During electrophoresis, the proteins were separated by molecular weight through the stacking and separation matrices at 250 volts for 40–50 min and then immobilized on the capillary wall, using proprietary photo-activated capture chemistry. The matrices were then washed out. The capillaries were next incubated with a blocking reagent for 15 min, and the target proteins were immunoprobed with primary antibodies followed by HRP-conjugated secondary antibodies (Anti-Rabbit IgG HRP, A00098, 10 μg/mL, GenScript, Piscataway, NJ, USA). The antibodies of GAPDH (NB300–322, 1:100, Novus Biologicals, Littleton, CO, USA) and Cx43 (C6219, 1:100, Sigma-Aldrich, St. Louis, MO, USA) were diluted in an antibody diluent (ProteinSimple) [25].

### 4.5. Data Analysis

All experiments in this study were designed with equally sized animal groups (*n* = 6), without carrying out a formal power analysis, in keeping with previous studies. All values are expressed as the mean ± SD, and *p* < 0.05 (two-tailed) was considered statistically significant for all tests. Drug levels in acute and subchronic administrations were selected based on values in previous studies. Where possible, we sought to randomize and blind the data. In particular, for the determination of transmitter levels and protein expression, the sample order on the autosampler and Wes were determined by a random number table.

Concentration-dependent effects of acute and subchronic administrations of antipsychotics on basal astroglial l-glutamate levels were analyzed by multivariate analysis of variance (MANOVA) using BellCurve for Excel ver. 3.2 (Social Survey Research Information Co., Ltd., Tokyo, Japan). When the data did not violate the assumption of sphericity (*p* > 0.05), the F-value of the MANOVA was analyzed, using sphericity-assumed degrees of freedom. However, if the assumption of sphericity was violated (*p* < 0.05), the F-value was analyzed, using Chi-Muller’s corrected degrees of freedom. When the F-value for the genotype/drug/time factors of MANOVA was significant, the data were analyzed by a Tukey’s multiple comparison test. The expression of Cx43 in the plasma membrane fraction was also analyzed by MANOVA with Tukey’s multiple comparison, using BellCurve for Excel.

## 5. Conclusions

The present study determined the concentration- and time-dependent effects of CLZ, QTP and BPZ on astroglial transmission of l-glutamate associated with Cx43, to explore the mechanisms of the mood-stabilizing antipsychotic actions of them. Neither acute and subchronic administrations of CLZ, QTP and BPZ affected basal astroglial l-glutamate release and Cx43 expression in the astroglial plasma membrane. After the activation of transcription of Cx43 induced by subchronic administration of therapeutic-relevant concentration of VPA, a histone deacetylase inhibitor, both acute and subchronic administrations of therapeutic-relevant concentration of CLZ increased astroglial l-glutamate release and Cx43 expression in the plasma membrane. Contrary to CLZ, both therapeutic-relevant concentration of QTP and BPZ acutely did not affect astroglial l-glutamate release or Cx43 expression in the plasma membrane, whereas subchronic administration of them enhanced both l-glutamate release and Cx43 expression. These results suggest that activation of astroglial hemichannel activities are probably involved in the action of mood-stabilizer and antipsychotics of these three antipsychotics. However, the mechanisms of upregulation of functional Cx43 containing astroglial hemichannel between CLZ, QTP and BPZ are not identical. The stimulatory effects of both CLZ and QTP on Cx43 were Akt signaling sensitive, but that of BPZ was Akt insensitive. Furthermore, CLZ activated Akt signaling within hours order, but the onset of QTP-induced Aky signaling activation required more than days order. Based on these discrepant mechanisms on Akt signaling among CLZ, QTP and BPZ can explain the adverse reactions associated with impaired glucose tolerance among CLZ, QTP and BPZ.

## Figures and Tables

**Table 1:** Summary of the concentration-dependent effects of acute and subchronic administrations of CLZ, QTP and BPZ on astroglial l-glutamate release and Cx43 expression in the plasma membrane.

	Administration	CLZ	QTP	BPZ	Figure
Astroglial glutamate release					
Basal glutamate release	Acute (120 min)	↑	→	→	Figure 1
	Subchronic (7 days)	→	→	→	Figure 1
Evoked glutamate release	Acute (120 min)	↑↑	↑	↑	Figure 2
	Subchronic (7 days)	↑↑	↑↑	↑↑	Figure 3
Evoked release	Acute (120 min)	↑↑	↑↑	↑↑	Figure 2
(+subchronic VPA)	Subchronic (7 days)	↑↑	↑↑	↑↑	Figure 3
Akt inhibitor sensitivity of glutamate release					
Evoked release	Acute (120 min)	↓	→	→	Figure 5
(+ subchronic VPA)	Subchronic (7 days)	↓	↓	→	Figure 5
Cx43 expression in the astroglial plasma membrane					
(+ subchronic VPA)	Acute (120 min)	↑↑	→	→	Figure 7
(+ subchronic VPA)	Subchronic (7 days)	↑↑	↑↑	↑↑	Figure 7
Akt sensitivity of Cx43 expression in the astroglial plasma membrane					
(+ subchronic VPA)	Acute (120 min)	↓↓	→	→	Figure 7
(+ subchronic VPA)	Subchronic (7 days)	↓↓	↓↓	→	Figure 7

→: no effect, ↑: increased by supratherapeutic concentration but unaffected by therapeutic-relevant concentration, ↑↑: increased by therapeutic-relevant concentration.

## Data Availability

The data presented in this study are available on request from the corresponding author. The data are not publicly available due to equipment dependent data.

## References

[1] Okubo R., Hasegawa T., Fukuyama K., Shiroyama T., Okada M. (2021). Current limitations and candidate potential of 5-ht7 receptor antagonism in psychiatric pharmacotherapy. Front. Psychiatry.

[2] Okada M., Oka T., Nakamoto M., Fukuyama K., Shiroyama T. (2020). Astroglial connexin43 as a potential target for a mood stabiliser. Int. J. Mol. Sci..

[3] Okada M., Fukuyama K., Shiroyama T., Murata M. (2020). A working hypothesis regarding identical pathomechanisms between clinical efficacy and adverse reaction of clozapine via the activation of connexin43. Int. J. Mol. Sci..

[4] Meltzer H.Y., Li Z., Kaneda Y., Ichikawa J. (2003). Serotonin receptors: Their key role in drugs to treat schizophrenia. Prog. Neuro Psychopharmacol. Biol. Psychiatry.

[5] Meltzer H.Y., Massey B.W. (2011). The role of serotonin receptors in the action of atypical antipsychotic drugs. Curr. Opin. Pharmacol..

[6] Okada M., Kawano Y., Fukuyama K., Motomura E., Shiroyama T. (2020). Candidate strategies for development of a rapid-acting antidepressant class that does not result in neuropsychiatric adverse effects: Prevention of ketamine-induced neuropsychiatric adverse reactions. Int. J. Mol. Sci..

[7] Okada M., Matsumoto R., Yamamoto Y., Fukuyama K. (2021). Effects of subchronic administrations of vortioxetine, lurasidone, and escitalopram on thalamocortical glutamatergic transmission associated with serotonin 5-ht7 receptor. Int. J. Mol. Sci.

[8] Fukuyama K., Ueda Y., Okada M. (2020). Effects of carbamazepine, lacosamide and zonisamide on gliotransmitter release associated with activated astroglial hemichannels. Pharmaceuticals.

[9] Fukuyama K., Okubo R., Murata M., Shiroyama T., Okada M. (2020). Activation of astroglial connexin is involved in concentration-dependent double-edged sword clinical action of clozapine. Cells.

[10] Fukuyama K., Okada M. (2020). Age-dependent and sleep/seizure-induced pathomechanisms of autosomal dominant sleep-related hypermotor epilepsy. Int. J. Mol. Sci..

[11] Fukuyama K., Fukuzawa M., Ruri O., Okada M. (2020). Upregulated connexin 43 induced by loss-of-functional s284l-mutant alpha4 subunit of nicotinic ach receptor contributes to pathomechanisms of autosomal dominant sleep-related hypermotor epilepsy. Pharmaceuticals.

[12] Fukuyama K., Fukuzawa M., Okada M. (2020). Upregulated and hyperactivated thalamic connexin 43 plays important roles in pathomechanisms of cognitive impairment and seizure of autosomal dominant sleep-related hypermotor epilepsy with s284l-mutant α4 subunit of nicotinic ach receptor. Pharmaceuticals.

[13] Okada M., Fukuyama K., Shiroyama T., Ueda Y. (2019). Carbamazepine attenuates astroglial l-glutamate release induced by pro-inflammatory cytokines via chronically activation of adenosine a2a receptor. Int. J. Mol. Sci..

[14] Okada M., Fukuyama K., Kawano Y., Shiroyama T., Ueda Y. (2019). Memantine protects thalamocortical hyper-glutamatergic transmission induced by nmda receptor antagonism via activation of system xc−. Pharmacol. Res. Perspect..

[15] Nakano T., Hasegawa T., Suzuki D., Motomura E., Okada M. (2019). Amantadine combines astroglial system xc− activation with glutamate/nmda receptor inhibition. Biomolecules.

[16] Fukuyama K., Kato R., Murata M., Shiroyama T., Okada M. (2019). Clozapine normalizes a glutamatergic transmission abnormality induced by an impaired nmda receptor in the thalamocortical pathway via the activation of a group iii metabotropic glutamate receptor. Biomolecules.

[17] Fukuyama K., Okada M. (2018). Effects of levetiracetam on astroglial release of kynurenine-pathway metabolites. Br. J. Pharmacol..

[18] Fukuyama K., Hasegawa T., Okada M. (2018). Cystine/glutamate antiporter and aripiprazole compensate nmda antagonist-induced dysfunction of thalamocortical l-glutamatergic transmission. Int. J. Mol. Sci..

[19] Fukuyama K., Tanahashi S., Hoshikawa M., Shinagawa R., Okada M. (2014). Zonisamide regulates basal ganglia transmission via astroglial kynurenine pathway. Neuropharmacology.

[20] Yamamura S., Hoshikawa M., Dai K., Saito H., Suzuki N., Niwa O., Okada M. (2013). Ono-2506 inhibits spike-wave discharges in a genetic animal model without affecting traditional convulsive tests via gliotransmission regulation. Br. J. Pharmacol..

[21] Tanahashi S., Yamamura S., Nakagawa M., Motomura E., Okada M. (2012). Clozapine, but not haloperidol, enhances glial d-serine and l-glutamate release in rat frontal cortex and primary cultured astrocytes. Br. J. Pharmacol..

[22] Okada M. (2021). Can rodent models elucidate pathomechanisms of genetic epilepsy?. Br. J. Pharmacol..

[23] Jeanson T., Pondaven A., Ezan P., Mouthon F., Charveriat M., Giaume C. (2015). Antidepressants impact connexin 43 channel functions in astrocytes. Front. Cell. Neurosci..

[24] Liu X., Gangoso E., Yi C., Jeanson T., Kandelman S., Mantz J., Giaume C. (2016). General anesthetics have differential inhibitory effects on gap junction channels and hemichannels in astrocytes and neurons. Glia.

[25] Fukuyama K., Fukuzawa M., Shiroyama T., Okada M. (2020). Pathogenesis and pathophysiology of autosomal dominant sleep-related hypermotor epilepsy with s284l-mutant alpha4 subunit of nicotinic ach receptor. Br. J. Pharmacol..

[26] Sun J.-D., Liu Y., Yuan Y.-H., Li J., Chen N.-H. (2012). Gap junction dysfunction in the prefrontal cortex induces depressive-like behaviors in rats. Neuropsychopharmacology.

[27] Orellana J.A., Moraga-Amaro R., Diaz-Galarce R., Rojas S., Maturana C.J., Stehberg J., Saez J.C. (2015). Restraint stress increases hemichannel activity in hippocampal glial cells and neurons. Front. Cell. Neurosci..

[28] Schoenfeld T.J., Kloth A.D., Hsueh B., Runkle M.B., Kane G.A., Wang S.S., Gould E. (2014). Gap junctions in the ventral hippocampal-medial prefrontal pathway are involved in anxiety regulation. J. Neurosci. Off. J. Soc. Neurosci..

[29] Fukuyama K., Fukuzawa M., Shiroyama T., Okada M. (2020). Pathomechanism of nocturnal paroxysmal dystonia in autosomal dominant sleep-related hypermotor epilepsy with s284l-mutant α4 subunit of nicotinic ach receptor. Biomed. Pharmacother..

[30] Bernard R., Kerman I.A., Thompson R.C., Jones E.G., Bunney W.E., Barchas J.D., Schatzberg A.F., Myers R.M., Akil H., Watson S.J. (2011). Altered expression of glutamate signaling, growth factor, and glia genes in the locus coeruleus of patients with major depression. Mol. Psychiatry.

[31] Ernst C., Nagy C., Kim S., Yang J.P., Deng X., Hellstrom I.C., Choi K.H., Gershenfeld H., Meaney M.J., Turecki G. (2011). Dysfunction of astrocyte connexins 30 and 43 in dorsal lateral prefrontal cortex of suicide completers. Biol. Psychiatry.

[32] Nagy C., Torres-Platas S.G., Mechawar N., Turecki G. (2017). Repression of astrocytic connexins in cortical and subcortical brain regions and prefrontal enrichment of h3k9me3 in depression and suicide. Int. J. Neuropsychopharmacol..

[33] Miguel-Hidalgo J.J., Wilson B.A., Hussain S., Meshram A., Rajkowska G., Stockmeier C.A. (2014). Reduced connexin 43 immunolabeling in the orbitofrontal cortex in alcohol dependence and depression. J. Psychiatr. Res..

[34] Okada M., Fukuyama K., Shiroyama T., Ueda Y. (2019). Lurasidone inhibits nmda antagonist-induced functional abnormality of thalamocortical glutamatergic transmission via 5-ht7 receptor blockade. Br. J. Pharmacol..

[35] Okada M., Fukuyama K., Okubo R., Shiroyama T., Ueda Y. (2019). Lurasidone sub-chronically activates serotonergic transmission via desensitization of 5-ht1a and 5-ht7 receptors in dorsal raphe nucleus. Pharmaceuticals.

[36] Okada M., Fukuyama K., Kawano Y., Shiroyama T., Suzuki D., Ueda Y. (2019). Effects of acute and sub-chronic administrations of guanfacine on catecholaminergic transmissions in the orbitofrontal cortex. Neuropharmacology.

[37] Okada M., Fukuyama K., Nakano T., Ueda Y. (2019). Pharmacological discrimination of effects of mk801 on thalamocortical, mesothalamic, and mesocortical transmissions. Biomolecules.

[38] Miguel-Hidalgo J.J., Moulana M., Deloach P.H., Rajkowska G. (2018). Chronic unpredictable stress reduces immunostaining for connexins 43 and 30 and myelin basic protein in the rat prelimbic and orbitofrontal cortices. Chronic Stress.

[39] Jin C., Wang Z.Z., Zhou H., Lou Y.X., Chen J., Zuo W., Tian M.T., Wang Z.Q., Du G.H., Kawahata I. (2017). Ginsenoside rg1-induced antidepressant effects involve the protection of astrocyte gap junctions within the prefrontal cortex. Prog. Neuro Psychopharmacol. Biol. Psychiatry.

[40] Lou Y.X., Wang Z.Z., Xia C.Y., Mou Z., Ren Q., Liu D.D., Zhang X., Chen N.H. (2020). The protective effect of ginsenoside rg1 on depression may benefit from the gap junction function in hippocampal astrocytes. Eur. J. Pharmacol..

[41] Quesseveur G., Portal B., Basile J.A., Ezan P., Mathou A., Halley H., Leloup C., Fioramonti X., Deglon N., Giaume C. (2015). Attenuated levels of hippocampal connexin 43 and its phosphorylation correlate with antidepressant- and anxiolytic-like activities in mice. Front. Cell. Neurosci..

[42] Miguel-Hidalgo J.J., Carter K., Deloach P.H., Sanders L., Pang Y. (2019). Glucocorticoid-induced reductions of myelination and connexin 43 in mixed central nervous system cell cultures are prevented by mifepristone. Neuroscience.

[43] Fatemi S.H., Folsom T.D., Reutiman T.J., Pandian T., Braun N.N., Haug K. (2008). Chronic psychotropic drug treatment causes differential expression of connexin 43 and gfap in frontal cortex of rats. Schizophr. Res..

[44] Morioka N., Suekama K., Zhang F.F., Kajitani N., Hisaoka-Nakashima K., Takebayashi M., Nakata Y. (2014). Amitriptyline up-regulates connexin43-gap junction in rat cultured cortical astrocytes via activation of the p38 and c-fos/ap-1 signalling pathway. Br. J. Pharmacol..

[45] Rajkowska G., Miguel-Hidalgo J.J., Wei J., Dilley G., Pittman S.D., Meltzer H.Y., Overholser J.C., Roth B.L., Stockmeier C.A. (1999). Morphometric evidence for neuronal and glial prefrontal cell pathology in major depression. Biol. Psychiatry.

[46] Ongur D., Drevets W.C., Price J.L. (1998). Glial reduction in the subgenual prefrontal cortex in mood disorders. Proc. Natl. Acad. Sci. USA.

[47] Cotter D., Mackay D., Chana G., Beasley C., Landau S., Everall I.P. (2002). Reduced neuronal size and glial cell density in area 9 of the dorsolateral prefrontal cortex in subjects with major depressive disorder. Cereb. Cortex.

[48] Bowley M.P., Drevets W.C., Ongur D., Price J.L. (2002). Low glial numbers in the amygdala in major depressive disorder. Biol. Psychiatry.

[49] Chana G., Landau S., Beasley C., Everall I.P., Cotter D. (2003). Two-dimensional assessment of cytoarchitecture in the anterior cingulate cortex in major depressive disorder, bipolar disorder, and schizophrenia: Evidence for decreased neuronal somal size and increased neuronal density. Biol. Psychiatry.

[50] Maes M., Yirmyia R., Noraberg J., Brene S., Hibbeln J., Perini G., Kubera M., Bob P., Lerer B., Maj M. (2009). The inflammatory & neurodegenerative (i&nd) hypothesis of depression: Leads for future research and new drug developments in depression. Metab. Brain Dis..

[51] Rajkowska G., Selemon L.D., Goldman-Rakic P.S. (1998). Neuronal and glial somal size in the prefrontal cortex: A postmortem morphometric study of schizophrenia and huntington disease. Arch. Gen. Psychiatry.

[52] Selemon L.D., Rajkowska G., Goldman-Rakic P.S. (1998). Elevated neuronal density in prefrontal area 46 in brains from schizophrenic patients: Application of a three-dimensional, stereologic counting method. J. Comparat. Neurol..

[53] Gottlicher M., Minucci S., Zhu P., Kramer O.H., Schimpf A., Giavara S., Sleeman J.P., Lo Coco F., Nervi C., Pelicci P.G. (2001). Valproic acid defines a novel class of hdac inhibitors inducing differentiation of transformed cells. EMBO J..

[54] Kishi T., Ikuta T., Matsuda Y., Sakuma K., Okuya M., Mishima K., Iwata N. (2020). Mood stabilizers and/or antipsychotics for bipolar disorder in the maintenance phase: A systematic review and network meta-analysis of randomized controlled trials. Mol. Psychiatry.

[55] Kishi T., Sakuma K., Okuya M., Matsuda Y., Esumi S., Hashimoto Y., Hatano M., Miyake N., Miura I., Mishima K. (2021). Effects of a conventional mood stabilizer alone or in combination with second-generation antipsychotics on recurrence rate and discontinuation rate in bipolar i disorder in the maintenance phase: A systematic review and meta-analysis of randomized, placebo-controlled trials. Bipolar Disord..

[56] Azorin J.M., Simon N. (2019). Dopamine receptor partial agonists for the treatment of bipolar disorder. Drugs.

[57] Vieta E., Sachs G., Chang D., Hellsten J., Brewer C., Peters-Strickland T., Hefting N. (2021). Two randomized, double-blind, placebo-controlled trials and one open-label, long-term trial of brexpiprazole for the acute treatment of bipolar mania. J. Psychopharmacol..

[58] Yatham L.N., Kennedy S.H., Parikh S.V., Schaffer A., Bond D.J., Frey B.N., Sharma V., Goldstein B.I., Rej S., Beaulieu S. (2018). Canadian network for mood and anxiety treatments (canmat) and international society for bipolar disorders (isbd) 2018 guidelines for the management of patients with bipolar disorder. Bipolar Disord..

[59] Goodwin G.M., Haddad P.M., Ferrier I.N., Aronson J.K., Barnes T., Cipriani A., Coghill D.R., Fazel S., Geddes J.R., Grunze H. (2016). Evidence-based guidelines for treating bipolar disorder: Revised third edition recommendations from the british association for psychopharmacology. J. Psychopharmacol..

[60] Verdolini N., Hidalgo-Mazzei D., Murru A., Pacchiarotti I., Samalin L., Young A.H., Vieta E., Carvalho A.F. (2018). Mixed states in bipolar and major depressive disorders: Systematic review and quality appraisal of guidelines. Acta Psychiatr. Scand..

[61] Vasudev A., Chaudhari S., Sethi R., Fu R., Sandieson R.M., Forester B.P. (2018). A review of the pharmacological and clinical profile of newer atypical antipsychotics as treatments for bipolar disorder: Considerations for use in older patients. Drugs Aging.

[62] Ronaldson K.J., Fitzgerald P.B., Taylor A.J., Topliss D.J., Wolfe R., McNeil J.J. (2012). Rapid clozapine dose titration and concomitant sodium valproate increase the risk of myocarditis with clozapine: A case-control study. Schizophr. Res..

[63] Schoretsanitis G., Paulzen M., Unterecker S., Schwarz M., Conca A., Zernig G., Grunder G., Haen E., Baumann P., Bergemann N. (2018). Tdm in psychiatry and neurology: A comprehensive summary of the consensus guidelines for therapeutic drug monitoring in neuropsychopharmacology, update 2017; a tool for clinicians. World J. Biol. Psychiatry.

[64] Hiemke C., Bergemann N., Clement H.W., Conca A., Deckert J., Domschke K., Eckermann G., Egberts K., Gerlach M., Greiner C. (2018). Consensus guidelines for therapeutic drug monitoring in neuropsychopharmacology: Update 2017. Pharmacopsychiatry.

[65] Fasciani I., Temperan A., Perez-Atencio L.F., Escudero A., Martinez-Montero P., Molano J., Gomez-Hernandez J.M., Paino C.L., Gonzalez-Nieto D., Barrio L.C. (2013). Regulation of connexin hemichannel activity by membrane potential and the extracellular calcium in health and disease. Neuropharmacology.

[66] Kar R., Batra N., Riquelme M.A., Jiang J.X. (2012). Biological role of connexin intercellular channels and hemichannels. Arch. Biochem. Biophys..

[67] Okada M., Fukuyama K., Shiroyama T., Ueda Y. (2021). Brivaracetam prevents astroglial l-glutamate release associated with hemichannel through modulation of synaptic vesicle protein. Biomed. Pharmacother..

[68] Yoshida S., Yamamura S., Ohoyama K., Nakagawa M., Motomura E., Kaneko S., Okada M. (2010). Effects of valproate on neurotransmission associated with ryanodine receptors. Neurosci. Res..

[69] Kaneko S., Battino D., Andermann E., Wada K., Kan R., Takeda A., Nakane Y., Ogawa Y., Avanzini G., Fumarola C. (1999). Congenital malformations due to antiepileptic drugs. Epilepsy Res..

[70] Ribeiro-Rodrigues T.M., Martins-Marques T., Morel S., Kwak B.R., Girao H. (2017). Role of connexin 43 in different forms of intercellular communication—Gap junctions, extracellular vesicles and tunnelling nanotubes. J. Cell Sci..

[71] Mitterauer B. (2009). Loss of function of glial gap junctions may cause severe cognitive impairments in schizophrenia. Med. Hypotheses.

[72] Gawlik M., Wagner M., Pfuhlmann B., Stober G. (2016). The role of pannexin gene variants in schizophrenia: Systematic analysis of phenotypes. Eur. Arch. Psychiatry Clin. Neurosci..

[73] Huhn M., Nikolakopoulou A., Schneider-Thoma J., Krause M., Samara M., Peter N., Arndt T., Backers L., Rothe P., Cipriani A. (2019). Comparative efficacy and tolerability of 32 oral antipsychotics for the acute treatment of adults with multi-episode schizophrenia: A systematic review and network meta-analysis. Lancet.

[74] Tiihonen J., Mittendorfer-Rutz E., Majak M., Mehtala J., Hoti F., Jedenius E., Enkusson D., Leval A., Sermon J., Tanskanen A. (2017). Real-world effectiveness of antipsychotic treatments in a nationwide cohort of 29823 patients with schizophrenia. JAMA Psychiatry.

[75] Ostacher M., Ng-Mak D., Patel P., Ntais D., Schlueter M., Loebel A. (2018). Lurasidone compared to other atypical antipsychotic monotherapies for bipolar depression: A systematic review and network meta-analysis. World J. Biol. Psychiatry.

[76] Fornaro M., Carvalho A.F., Fusco A., Anastasia A., Solmi M., Berk M., Sim K., Vieta E., de Bartolomeis A. (2020). The concept and management of acute episodes of treatment-resistant bipolar disorder: A systematic review and exploratory meta-analysis of randomized controlled trials. J. Affect. Disord..

[77] Alper K., Schwartz K.A., Kolts R.L., Khan A. (2007). Seizure incidence in psychopharmacological clinical trials: An analysis of food and drug administration (fda) summary basis of approval reports. Biol. Psychiatry.

[78] Wu C.S., Wang S.C., Yeh I.J., Liu S.K. (2016). Comparative risk of seizure with use of first- and second-generation antipsychotics in patients with schizophrenia and mood disorders. J. Clin. Psychiatry.

[79] Walrave L., Vinken M., Leybaert L., Smolders I. (2020). Astrocytic connexin43 channels as candidate targets in epilepsy treatment. Biomolecules.

[80] Hussein A.M., Ghalwash M., Magdy K., Abulseoud O.A. (2016). Beta lactams antibiotic ceftriaxone modulates seizures, oxidative stress and connexin 43 expression in hippocampus of pentylenetetrazole kindled rats. J. Epilepsy Res..

[81] Das A., Wallace G.C., Holmes C., McDowell M.L., Smith J.A., Marshall J.D., Bonilha L., Edwards J.C., Glazier S.S., Ray S.K. (2012). Hippocampal tissue of patients with refractory temporal lobe epilepsy is associated with astrocyte activation, inflammation, and altered expression of channels and receptors. Neuroscience.

[82] Garbelli R., Frassoni C., Condorelli D.F., Trovato Salinaro A., Musso N., Medici V., Tassi L., Bentivoglio M., Spreafico R. (2011). Expression of connexin 43 in the human epileptic and drug-resistant cerebral cortex. Neurology.

[83] Tanahashi S., Yamamura S., Nakagawa M., Motomura E., Okada M. (2012). Dopamine d2 and serotonin 5-ht1a receptors mediate the actions of aripiprazole in mesocortical and mesoaccumbens transmission. Neuropharmacology.

[84] Jeon S.M., Park S., Kim D., Kwon J.W. (2021). Risk of seizures associated with antipsychotic treatment in pediatrics with psychiatric disorders: A nested case-control study in korea. Eur. Child Adolesc. Psychiatry.

[85] Jakobsen K.D., Bruhn C.H., Pagsberg A.K., Fink-Jensen A., Nielsen J. (2016). Neurological, metabolic, and psychiatric adverse events in children and adolescents treated with aripiprazole. J. Clin. Psychopharmacol..

[86] Hou J.C., Pessin J.E. (2007). Ins (endocytosis) and outs (exocytosis) of glut4 trafficking. Curr. Opin. Cell Biol..

[87] Emamian E.S., Hall D., Birnbaum M.J., Karayiorgou M., Gogos J.A. (2004). Convergent evidence for impaired akt1-gsk3beta signaling in schizophrenia. Nat. Genet..

[88] Pirola L., Bonnafous S., Johnston A.M., Chaussade C., Portis F., Van Obberghen E. (2003). Phosphoinositide 3-kinase-mediated reduction of insulin receptor substrate-1/2 protein expression via different mechanisms contributes to the insulin-induced desensitization of its signaling pathways in l6 muscle cells. J. Biol. Chem..

[89] Panariello F., Perruolo G., Cassese A., Giacco F., Botta G., Barbagallo A.P., Muscettola G., Beguinot F., Formisano P., de Bartolomeis A. (2012). Clozapine impairs insulin action by up-regulating akt phosphorylation and ped/pea-15 protein abundance. J. Cell. Physiol..

[90] Frampton J.E. (2019). Brexpiprazole: A review in schizophrenia. Drugs.

[91] Maeda K., Sugino H., Akazawa H., Amada N., Shimada J., Futamura T., Yamashita H., Ito N., McQuade R.D., Mork A. (2014). Brexpiprazole i: In vitro and in vivo characterization of a novel serotonin-dopamine activity modulator. J. Pharmacol. Exp. Ther..

[92] Beaulieu J.M., Sotnikova T.D., Yao W.D., Kockeritz L., Woodgett J.R., Gainetdinov R.R., Caron M.G. (2004). Lithium antagonizes dopamine-dependent behaviors mediated by an akt/glycogen synthase kinase 3 signaling cascade. Proc. Natl. Acad. Sci. USA.

[93] Beaulieu J.M., Sotnikova T.D., Marion S., Lefkowitz R.J., Gainetdinov R.R., Caron M.G. (2005). An akt/beta-arrestin 2/pp2a signaling complex mediates dopaminergic neurotransmission and behavior. Cell.

